# Psychophysiological Responses to Group Exercise Training Sessions: Does Exercise Intensity Matter?

**DOI:** 10.1371/journal.pone.0149997

**Published:** 2016-08-04

**Authors:** Matteo Vandoni, Erwan Codrons, Luca Marin, Luca Correale, Marcelo Bigliassi, Cosme Franklim Buzzachera

**Affiliations:** 1 Department of Public Health, Experimental Medicine & Forensic Science, University of Pavia, Pavia, Italy; 2 Department of Brain and Behavioral Sciences, University of Pavia, Pavia, Italy; 3 Department of Physical Education, State University of Parana, Londrina, Brazil; 4 Department of Physical Education, North University of Parana, Londrina, Brazil; Texas A&M University, UNITED STATES

## Abstract

Group exercise training programs were introduced as a strategy for improving health and fitness and potentially reducing dropout rates. This study examined the psychophysiological responses to group exercise training sessions. Twenty-seven adults completed two group exercise training sessions of moderate and vigorous exercise intensities in a random and counterbalanced order. The %HRR and the exertional and arousal responses to vigorous session were higher than those during the moderate session (*p*<0.05). Consequently, the affective responses to vigorous session were less pleasant than those during moderate session (*p*<0.05). These results suggest that the psychophysiological responses to group exercise training sessions are intensity-dependent. From an adherence perspective, interventionists are encouraged to emphasize group exercise training sessions at a moderate intensity to maximize affective responses and to minimize exertional responses, which in turn may positively affect future exercise behavior.

## Introduction

Though it is well known that regular exercise promotes health and fitness benefits [1], unfortunately, most adults do not meet the minimum recommended levels of exercise participation [2]. Epidemiological data indicate that more than 60% of the worldwide population does not engage in either moderate physical activity for ≥ 30 min at least five times a week or vigorous physical activity for ≥ 20 min at least three times a week [2]. Dropout is considered one of the major contributing factors to the low rates of exercise participation. Evidence shows that approximately 50% of individuals who initiate an exercise program drop out within the first few months of participation [3]. Thus, many people withdraw from exercise programs before physiological gains occur. The identification of potential factors that contribute to non-adherence has been one of the greatest challenges in exercise research over the last few years.

The prescription of vigorous-intensity activities has been recognized as a contributing factor to non-adherence to exercise programs. The current recommendations developed by the American College of Sports Medicine confirmed that adherence is lower in higher-intensity exercise programs. Data from both epidemiological and intervention studies have also shown that sedentary individuals are more likely to adhere to low-intensity activities than to high-intensity activities [3,4,5]. These studies support the notion that exercise intensity influences the adherence to exercise programs. However, none of these investigations provide any information about potential mediators of the relationship between exercise intensity and adherence.

There is speculation that the relationship between exercise intensity and adherence to an exercise program might be mediated by the amount of pleasure that an individual experiences during exercise [6,7,8]. A positive affective response experienced during exercise may lead to greater enjoyment of the activity, creation of a positive memory of the exercise experience, and possibly increased motivation for engagement in future physical activity [3,8]. This hypothesis has been presented using the so-called hedonic theory of motivation [9], which argues that if people derive pleasure from exercise participation, then they would presumably seek to repeat this activity. Conversely, if people derive displeasure from exercise participation, the chances of them repeating this activity would be reduced. In fact, a recent study conducted by Williams et al. [8] reported that affective responses to a moderately intense stimulus were predictive of exercise participation up to 6 and 12 months later. However, the relationship between affective responses and later exercise participation becomes nonsignificant when controlling for perceived exertion, thus suggesting a crucial role for both affective and exertional responses in the adherence to exercise programs.

Group exercise training programs have been introduced in many fitness centers around the world. These programs involve exercising the major muscle groups using weights and/or bodyweight, a standard sequence of music selections, and choreography that is followed by the practitioners. One of the main purposes of these sessions is to improve the health and fitness of the practitioners. Studies utilizing this type of activity have shown that, on average, individuals are predisposed to exercise at intensities that lie within the range recommended to provide health and fitness benefits by the American College of Sports Medicine [10,11]. However, none of these studies provided any information about the affective and exertional responses to group exercise training programs, which may play a key role in determining the adherence to such an activity [8]. Another unsolved issue is whether the psychophysiological responses to this type of activity vary across the range of exercise intensities. Therefore, the purpose of this study was to examine the psychophysiological responses to group exercise training sessions performed at moderate and vigorous intensities. Based upon previous data and theories, we hypothesized that a moderate-intensity exercise trial would result in a more positive affective valence and a less strenuous perceived exertion compared with a vigorous-intensity exercise trial.

## Material and Methods

### Study Design

To examine our hypothesis, a group of active, young men and women completed four trials, scheduled on different days, with at least 48–72 h between trials. On Day 1, participants underwent a medical screening, anthropometric measurement, and familiarization with the experimental procedures. On Day 2, they performed a maximal graded exercise test. Finally, on Days 3–4, participants performed two group exercise training sessions at moderate and vigorous intensities, in a random and counterbalanced order. This study design enables for the direct comparison of the effects of exercise intensity and gender (independent variables) on psychophysiological responses to group exercise training sessions (dependent variables: %HRR, perceived exertion, affective valence, and perceived activation).

### Participants

Twenty-seven individuals (17 men and 10 women) between 18 and 28 years of age volunteered to participate in this study, which was performed in accordance with the Helsinki Declaration of 1975 and approved by the Institutional Ethics Committee. Each participant gave their written informed consent after the purpose, experimental procedures, possible risks, and benefits of the study were explained to them. The participants received no monetary compensation but were given the results of their maximal exercise test and an individualized exercise prescription upon completion of the study. Based on a power analysis considering the correlation among measures [12], the a priori sample size was calculated using a software package, GPower, [13]. This power analysis was conducted as follows: 2 (exercise intensity trials) × 5 (time), within subject factorial design with repeated measures, with effect size estimates derived from previous work [14]. For this study design, 6 participants were required for each of the cells to result in a statistical power of 0.99, with a large effect size, *f*^*2*^ = 1.00, [15], a moderate correlation (*r* = 0.50), and an overall level of significance of *p* = 0.05. In sum, the sample size of 27 participants was large enough to detect the expected differences between exercise intensity trials.

Participants were recruited from exercise and health courses at the University. However, to be eligible for participation, individuals were required to have engaged in > 30 min of moderate physical activity per day on most days of the week for the previous six months [1]. All participants were in good health; were not taking any medications known to affect cardiovascular, respiratory, muscular, metabolic or cognitive functions; had a stable body mass (<2.5-kg net change over the previous 3 months); and were nonsmokers. The participants’ descriptive data are shown in Table 1.

**Table 1:** Descriptive data of the participants.

	Men (n = 17)	Women (n = 10)
Age (years)	22.5 ± 2.0	23.3 ± 2.2
Weight (kg)	74.7 ± 7.7	55.3 ± 4.1 *
Height (cm)	178.8 ± 4.8	167.8 ± 6.0 *
BMI (kg.m^-2^)	23.3 ± 2.3	19.4 ± 1.2 *
HR_Rest_ (beats · min^-1^)	69.5 ± 13.2	70.9 ± 13.0
HR_max_ (beats · min^-1^)	183.2 ± 9.1	178.2 ± 15.3
VT (%VO_2max_)	46.2 ± 7.3	47.1 ± 10.4
VO_2max_ (mL · kg^-1^. min^-1^)	51.3 ± 6.2	37.4 ± 8.0 *

*Note*. Values are mean ± SD.

* *p* < 0.01, statistically significant difference. BMI, body mass index; HR_Rest_, Resting heart rate; HR_max_, maximal heart rate; VT, ventilatory threshold; VO_2max_, maximal oxygen consumption.

### Procedures

#### Experimental Trials

All participants completed two experimental trials, which were scheduled on different days and with at least 48–72 h between trials. Specifically, the participants performed two sessions of group exercise training, involving selected bodyweight exercises targeting the major muscle groups and performed according to music and choreography sequences. All sessions were structured as follows: 15 min of standard warm up with no music, 60 min of functional exercises with synchronous music, and 15 min of cool down with no music. As noted by Simpson and Karageorghis [16], the use of synchronous music entails the conscious performance of repetitive movements in time with the rhythmical elements of music, such as the beat or tempo. In the present study, the trials were performed using synchronous medium-tempo (120 beats · min^-1^) or fast-tempo (145 beats · min^-1^) music, and participants were instructed to synchronize their body movements according to the music tempo and the trainer’s choreography. Therefore, the trials used in this study were described as moderate- and vigorous-intensity exercise trials, respectively. As proposed by Karageorghis et al. [17], the aforementioned synchronous tempo music may adequately lead to moderate-intensity (120 beats · min^-1^) and vigorous-intensity (145 beats · min^-1^) exercises. The music was played through wall-mounted speakers at a safe and effective standardized intensity [17] in each of the trials. To avoid any influence of different music on the psychophysiological responses to exercise, standardized music was used in the trials [17]. All participants were instructed to individually report their psychological responses to exercise to minimize any possible effects of group dynamics or social interactions. All participants performed the two experimental trials in a random and counterbalanced order. They were instructed to refrain from exercise, avoid alcoholic and caffeinated products in the 24 h before the trials, and present themselves at the laboratory in a 2-h post-absorptive state. All experimental trials were conducted in the morning (between 8:00 am and 12:00 pm) under similar environmental conditions.

HR was continuously recorded before and throughout the trials using a short-range radio telemetry HR monitor (Polar Team System, Polar Electro^™^, Kempele, Finland). The HR responses were then averaged for the final 30-sec intervals of every 15-min period of each trial and 1 min before and 1 min after exercise. For each participant, heart rate reserve (HRR) was calculated by subtracting the HR_Rest_ value from the respective maximal value (i.e., HR_max_). Accordingly, for each 15-min interval of each of the trials, as well as 1 min before and 1 min after exercise, the increment above resting for each value was divided by the calculated reserve and multiplied by 100 to derive %HRR [18].

#### Maximal Graded Exercise Test

After 5 min of seated rest, participants performed a maximal graded exercise test on a motorized treadmill. The standard Bruce protocol was used to determine maximal oxygen consumption (VO_2max_). Each participant was verbally encouraged to continue to exercise until the point of volitional exhaustion. To achieve a VO_2max_, participants were required to meet two of the following criteria: (a) a plateau in VO_2_ (changes of < 150 mL · min^-1^ in the last three consecutive 15 sec averages), (b) a respiratory exchange ratio of ≥ 1.15, and (c) a heart rate (HR) within 10 beats · min^-1^ of the age-predicted maximum. Therefore, VO_2max_ was defined as the highest value of VO_2_ attained after reaching the aforementioned criteria. HR_max_ was defined as the highest HR value recorded over any continuous 15-sec interval during the test, whereas HR_Rest_ was considered the average HR value recorded over the last 2 min of seated rest [18]. Ventilatory threshold (VT) was determined by two independent experienced investigators in a blind fashion using the ventilatory equivalent method [19, 20].

HR (beats · min^-1^) was continuously recorded before and throughout the test using an HR monitor (RS400, Polar Electro™, Kempele, Finland). VO_2_, carbon dioxide production (VCO_2_), and pulmonary ventilation (VE) were measured using a portable gas analysis system (K4b^2^, Cosmed^™^, Rome, Italy). The system was calibrated using room air (21% O_2_, 0.03% CO_2_) and a certified gas mixture (16% O_2_, 5% CO_2_; Scott Medical Products^™^, Plumsteadville, USA) prior to each test. The turbine flowmeter was periodically calibrated using a 3-L syringe according to the manufacturer’s instructions.

### Psychometric Measures

#### Perceived Exertion, Affective Valence, and Perceived Activation

The 6–20 Borg Rate of Perceived Exertion (RPE) scale [21] was used as a measure of whole-body perceived exertion during exercise. This scale is a 15-point single-item measure, ranging from 6 to 20, with verbal anchors ranging from “no exertion at all” (6) to “maximal exertion” (20). Participants were previously anchored to the scale during the orientation session using memory-anchoring procedures [22]. The ratings of perceived exertion were estimated at the end of each 3-min stage of the maximal graded exercise test and at every 15-min interval of the exercise intensity trials.

Affective valence was measured using the Feeling Scale (FS) [23]. The FS is a single-item indicator of affective valence scored on an 11-point scale ranging from -5 “very bad” to +5 “very good”. The “core” or “basic” affective valence measured by the FS is a major component of the circumplex model, presented by Russell et al. [24], which incorporates affective valence and activation as orthogonal and bipolar dimensions of the affective space. In the present study, the Felt Arousal Scale (FAS) [25] was used as a measure of perceived activation, which is considered a global index of “somatic” arousal. The FAS is a 6-point, single-item, bipolar measure, ranging from 1 to 6, with verbal anchors of “low arousal” (1) and “high arousal” (6). In a counterbalanced manner 6–20 Borg RPE, FS, and FAS scales were administered at the end of each 3-min stage of the maximal graded exercise test and at every 15-min interval of the exercise intensity trials. Additionally, these scales were administered at two points in the exercise intensity trial: (i) immediately after the warm up (0 min) and (ii) immediately after the cool down. Participants rated “how” and “what” they felt at these particular moments. Standard definitions of perceived exertion, affective valence, and perceived activation, along with separate instructional sets for the 6–20 Borg RPE, FS, and FAS scales, were read to the participants before the maximal graded exercise test and exercise intensity trials [6,22,25]. During the maximal graded exercise test, the 6–20 Borg RPE, FS, and FAS scales were in full view of the participants at all times of the test.

#### Self-Reported Preference for Exercise Intensity

Preference was defined as a predisposition to select a particular level of exercise intensity when given the opportunity [26]. The Preference for and Tolerance of the Intensity of Exercise Questionnaire (PRETIE-Q) was used as a measure of self-reported preference for exercise intensity. Developed by Ekkekakis et al. [26], the eight-item PRETIE-Q Preference Scale contains four items that tap preference for high intensity (e.g., “I would rather have a short, intense workout than a long, low-intensity workout”) and four that tap preference for low intensity and are reverse-scored (e.g., “when I exercise, I usually prefer a slow, steady pace”). Each item is scored on a 5-point response scale ranging from “I totally disagree” (1) to “I totally agree” (5). At the end of the orientation session, the participants were given copies of the eight-item PRETIE-Q Preference Scale and were asked to complete and return them upon arrival for the next session. Given the recent exercise experience among these participants, it was possible for them to provide meaningful responses to the questions of the scale, which inquires about usual responses to the bodily stimulation generated during exercise [27].

#### Self-Efficacy

Self-efficacy is a concept relevant only to specifically delineated tasks, so it is assessed using task-specific, rather than domain-general, measures [28]. However, given the myriad of situations in which self-efficacy is a concern, attempting to develop countless scales for every conceivable situation seems unrealistic [29]. In this study, the Physical Self Efficacy Scale (PSES) was used as a measure of two differentially meaningful components: perceived physical ability (PPA) and physical self-presentation confidence (PSPC). The PPA subscale appraises the perceptions of individuals’ competence in performing tasks that utilize physical skills, while the PSPC subscale measures their confidence as reflected by their physical demeanor in the presence of others [30]. Developed by Ryckman et al. [30], the PSES contains 10 items that tap PPA (e.g., “I have a strong grip”) and 12 items that tap PSPC (e.g., “I find that I am not accident prone”) and are reverse-scored. Each item is scored on a 6-point response scale ranging from “strongly agree” (1) to “strongly disagree” (6), with higher scores reflecting stronger PPA and PSPC. At the end of the orientation session, the participants were given copies of the 22-item PSES and were asked to complete and return them upon arrival for the next session.

### Statistical Analysis

Data are shown as means ± standard deviations or standard errors. Independent *t*-tests were used to examine gender differences in anthropometric characteristics and physiological responses during the graded exercise test (*p* < 0.05). A series of three-factor [gender (men and women) × exercise intensity trial (moderate and vigorous intensities) × time (0 min, 15 min, 30 min, 45 min, and cool down)] repeated measures analysis of variance (ANOVA) was conducted on %HRR, perceived exertion, affective valence, and perceived activation. Initially, gender was included as a between-subject factor in all analyses conducted on the dependent variables (i.e., %HRR, perceived exertion, affective valence, and perceived activation). However, none of the main or interaction effects of gender (e.g., gender × exercise intensity trial, gender × time, and gender × exercise intensity trial × time) were statistically significant. As a consequence, gender was omitted. Whenever the sphericity assumption was violated in the ANOVA models, degrees of freedom were adjusted using the Greenhouse-Geisser or Huynh Feldt epsilon corrections. Partial eta squared (η^2^_p_) was used to estimate the sizes of the effects [15]. For each ANOVA model with a significant exercise intensity trial × time interaction, the simple effects of time were further analyzed within each exercise intensity trial. Significant simple effects of time were followed by planned contrasts, in which 15 min, 30 min, 45 min, and cool down values were compared with their 0 min (i.e., end of warm up) value. Because these comparisons increase the risk for type I error (i.e., to reject the null hypothesis when it should not be rejected), the *p* value for *post hoc* analyses was adjusted according to the Bonferroni correction to 0.05/4 = 0.0125. Additionally, Pearson correlation *r* coefficients were calculated to examine the relationship of intensity preference and the self-efficacy components (i.e., PPA and PSPC) with the psychophysiological responses (i.e., %HRR, perceived exertion, affective valence, and perceived activation) for each of the two exercise intensity trials. All data were analyzed using SPSS 17.0 for Windows (SPSS, Inc., Chicago, USA).

## Results

Descriptive data for the participants are shown in Table 1. There were no gender differences in age, VT, HR_Rest_, and HR_max_. However, the men were significantly heavier and taller (*p* < 0.001) and had greater BMIs and VO_2max_ values than the women (*p* < 0.001).

**Fig 1:**
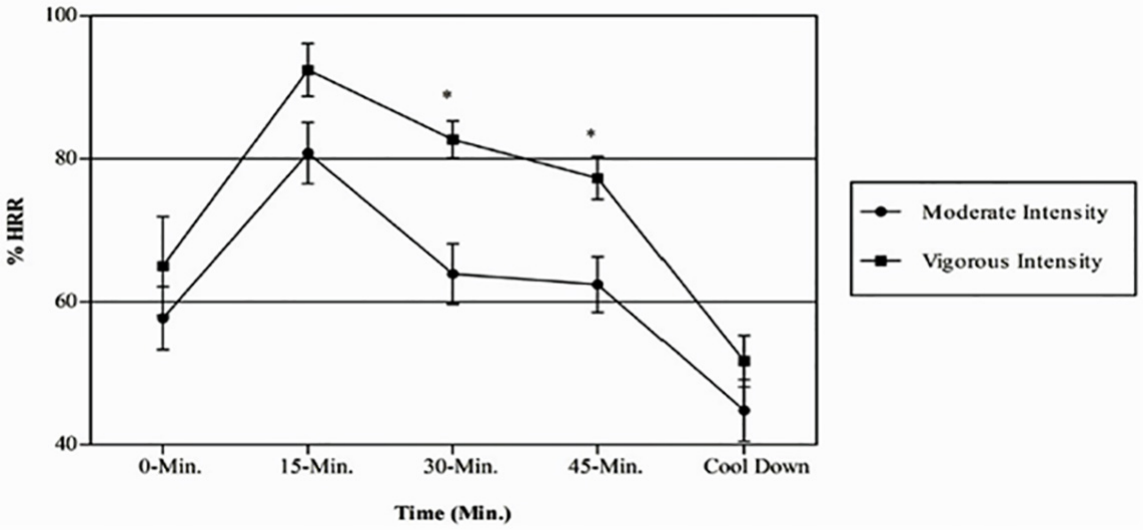
Physiological (%HRR) responses to the two exercise intensity trials. Data are shown as means ± standard error. * *p* < 0.01, moderate-exercise intensity significantly different from vigorous-exercise intensity.

The %HRR responses to the two different exercise intensity trials are presented in Fig 1. There were significant main effects of exercise intensity trial, *F*(1,23) = 5.788, *p* = 0.025, η^2^_p_ = 0.201, and time, *F*(2.659,61.155) = 122.740, *p* < 0.001, η^2^_p_ = 0.842. There was also a significant interaction between the exercise intensity trial and the time, *F*(2.028,46.646) = 4.073, *p* = 0.023, η^2^_p_ = 0.150. This finding demonstrates that the changes in %HRR responses to exercise differed as a function of the exercise intensity trial. Therefore, the interaction was decomposed into the simple effects of time within each exercise intensity trial. For the moderate-intensity trial, there was a significant effect of time, *F*(4,96) = 54.670, *p* < 0.001, η^2^_p_ = 0.695. Planned contrasts on the %HRR data indicated that the %HRR response was significantly lower at 0 min (i.e., end of warm up) compared with those at 15 min, 30 min, and 45 min (*p* < 0.0125), but was similar to that at cool down. There was also a significant effect of time for the vigorous-intensity trial, *F*(4,108) = 99.406, *p* < 0.001, η^2^_p_ = 0.786. Planned contrasts on the %HRR data indicated that the %HRR response was significantly lower at 0 min (i.e., end of warm up) compared with that at 15 min (*p* < 0.0125), and was similar to those at 30 min, 45 min, and cool down.

**Fig 2:**
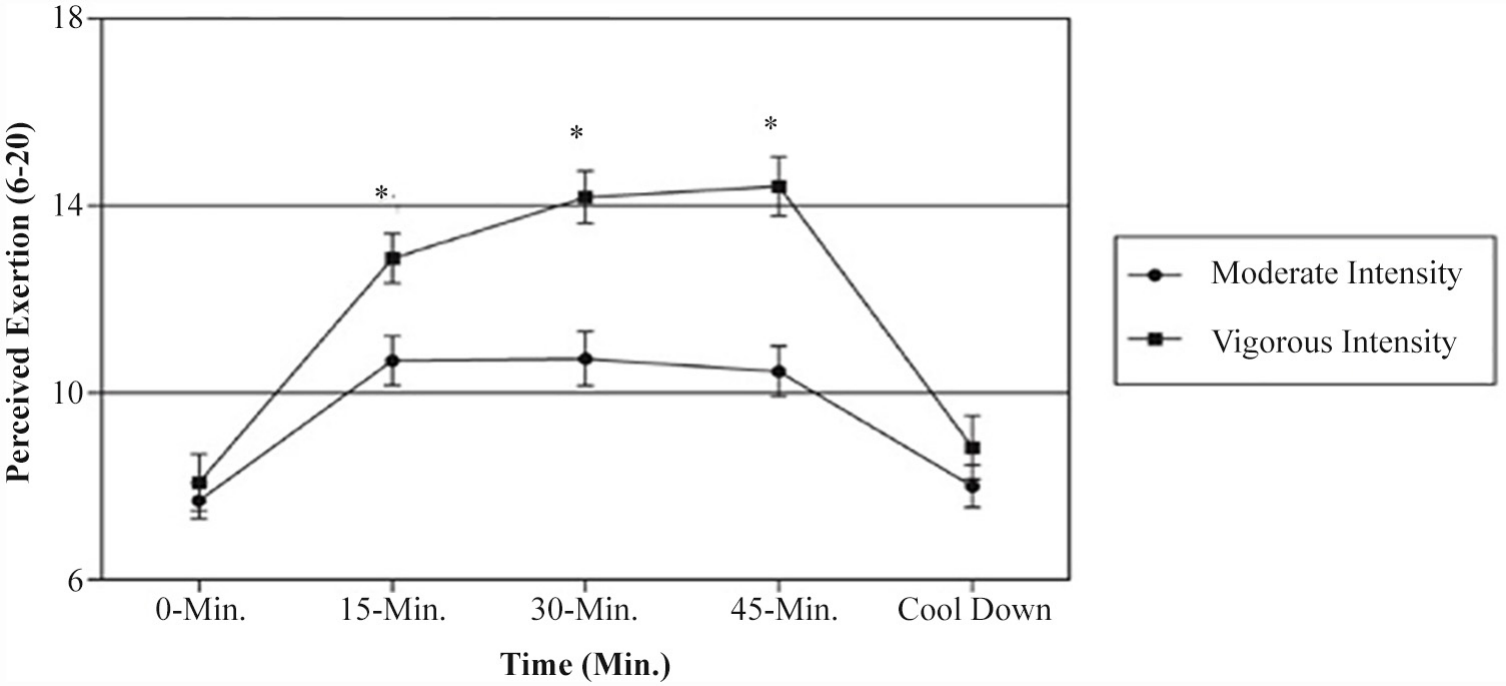
Perceived exertion responses to the two exercise intensity trials. Data are shown as means ± standard error. * *p* < 0.01, moderate-exercise intensity significantly different from vigorous-exercise intensity.

The exertional responses to the two different exercise intensity trials are presented in Fig 2. There were significant main effects of exercise intensity trial, *F*(1,26) = 18.238, *p* < 0.001, η^2^_p_ = 0.412, and time, *F*(2.955,71.634) = 147.761, *p* < 0.001, η^2^_p_ = 0.850. There was also a significant interaction between the exercise intensity trial and the time, *F*(2.643,68.725) = 15.123, *p* < 0.001, η^2^_p_ = 0.368. This finding demonstrates that the changes in perceived exertion during the exercise sessions differed as a function of the exercise intensity trial. Therefore, the interaction was decomposed into the simple effects of time within each exercise intensity trial. For the moderate-intensity trial, there was a significant effect of time, *F*(4,112) = 50.881, *p* < 0.001, η^2^_p_ = 0.645. Planned contrasts on the perceived exertion data indicated that the exertional response was significantly lower at 0 min (i.e., end of warm up) compared with those at 15 min, 30 min, and 45 min (*p* < 0.0125), but was similar to that at cool down. There was also a significant effect of time for the vigorous-intensity trial, *F*(4,104) = 92.092, *p* < 0.001, η^2^_p_ = 0.780. Planned contrasts on the perceived exertion data indicated significant increases in perceived exertion (in comparison with 0-min values) at 15 min, 30 min, and 45 min (*p* < 0.0125), but not at cool down. The exertional response was also significantly higher at 15 min compared with those at 30 min and 45 min (*p* < 0.0125).

**Fig 3:**
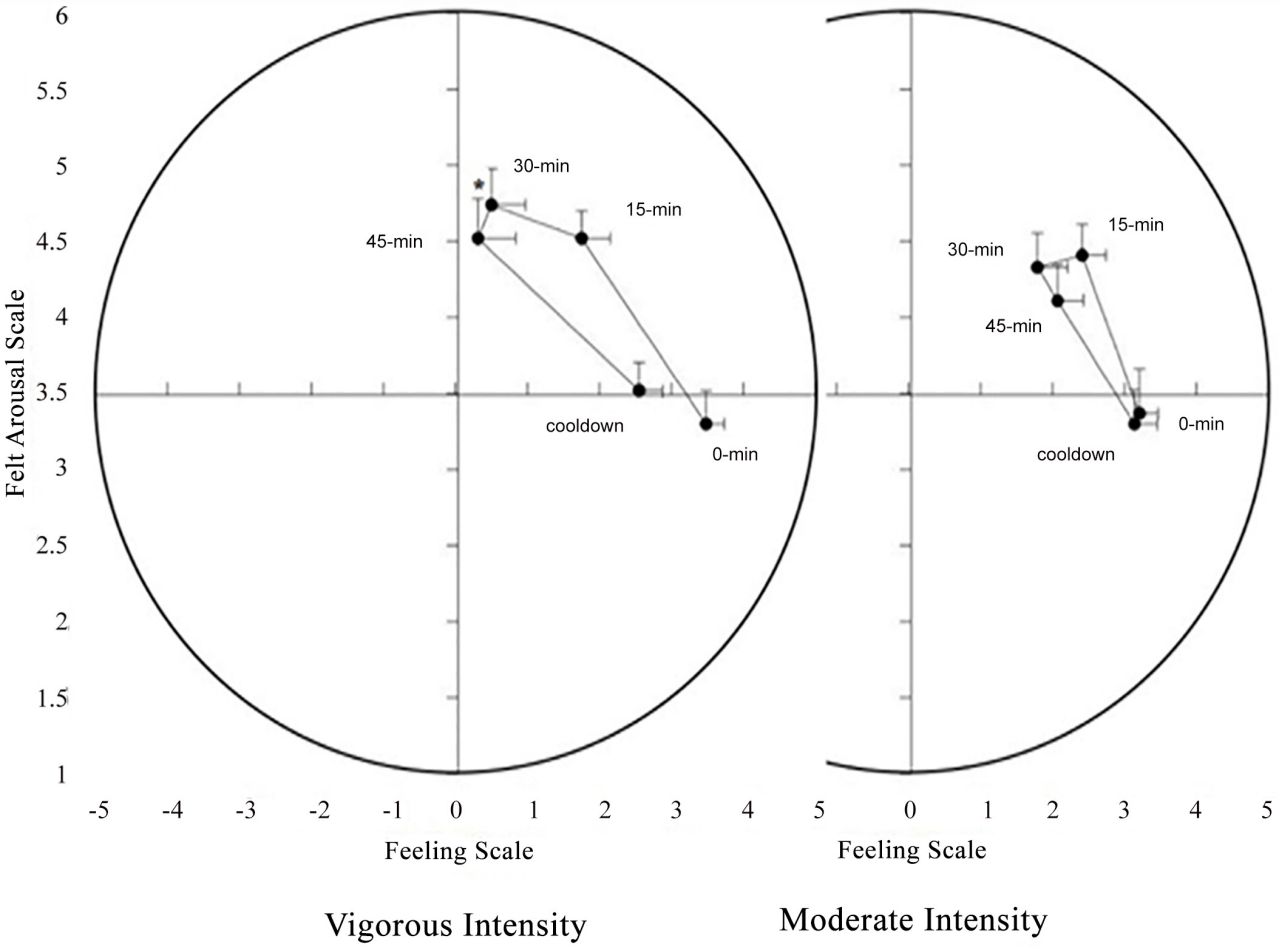
Temporal dynamics of the affective valence and perceived activation responses to the two exercise intensity trials. Data are shown as means ± standard error. * *p* < 0.01, moderate-exercise intensity significantly different from vigorous-exercise intensity.

The affective responses to the two different exercise intensity trials are presented in Fig 3. There were significant main effects of exercise intensity trial, *F*(1,26) = 3.808, *p* = 0.062, η^2^_p_ = 0.128, and time, *F*(2.207,57.386) = 25.717, *p* < 0.001, η^2^_p_ = 0.497. In particular, a more positive affective response occurred during the moderate-intensity trial than during the vigorous-intensity trial. However, there was a significant interaction between the exercise intensity trial and the time, *F*(2.427,63.090) = 5.343, *p* = 0.004, η^2^_p_ = 0.170. This finding demonstrates that the changes in affective responses to exercise differed as a function of the exercise intensity trial. Therefore, the interaction was decomposed into the simple effects of time within each exercise intensity trial. For the moderate-intensity trial, there was a significant effect of time, *F*(4,112) = 11.658, *p* < 0.001, η^2^_p_ = 0.294. Planned contrasts on the “core” affective valence data indicated that the affective response was significantly more positive at 0 min (i.e., end of warm up) compared with those at 15 min and 30 min (*p* < 0.0125), but was similar to those at 45 min and cool down. There was also a significant effect of time for the vigorous-intensity trial, *F*(4,104) = 17.775, *p* < 0.001, η^2^_p_ = 0.406. Planned contrasts on the “core” affective valence data indicated that the affective response was significantly more positive at 0 min (i.e., end of warm up) compared with those at 15 min, 30 min, and 45 min (*p* < 0.0125), but was similar to that at cool down in both trials.

The “somatic” arousal responses to the two different exercise intensity trials are presented in Fig 3. There was a significant main effect of the time *F*(1.731, 45.001) = 19.221, *p* < 0.001, η^2^_p_ = 0.425. In contrast, there was no significant main effect of exercise intensity trial (*p* = 0.60) or a significant exercise intensity trial × time interaction (*p* = 0.80). These findings demonstrate that the changes in perceived activation during the exercise sessions were similar for the two exercise intensity trials. As a result, the exercise intensity trials were collapsed for planned contrasts that compared the 0-min (i.e., end of warm up) value with the 15-min, 30-min, 45-min, and cool down values. These analyses indicated significant increases in perceived activation (compared with 0-min values) at 15 min and 30 min (*p* < 0.001), but not at 45 min and cool down, for the two exercise intensity trials.

The correlations between the intensity preference and self-efficacy components with the psychophysiological responses at each of the two exercise intensity trials are presented in Table 2. The correlation *r* coefficients for each variable were consistent across the five time points (i.e., 0 min, 15 min, 30 min, 45 min, and cool down) within each exercise intensity trial. This consistency is important because the stability of correlational statistics can be a point of concern with small sample sizes (e.g., < 100) [27]. The results indicate that there were no significant correlations of the intensity preference and the self-efficacy components with the psychophysiological responses in any of the two exercise intensity trials (*p* > 0.05) (Table 2).

**Table 2:** Pearson average correlation *r* coefficients of preference for and the self-efficacy components with psychophysiological responses at each of the two exercise intensity trials.

	Preference For	PPA	PSPC
Vigorous-exercise intensity Trial			
%HRR	-0.03	0.33	0.33
Perceived Exertion	-0.01	0.24	0.24
Affective Valence	-0.07	0.08	0.33
Perceived Activation	-0.01	0.06	0.24
Moderate-exercise intensity Trial			
%HRR	-0.03	-0.24	-0.27
Perceived Exertion	-0.02	-0.39	-0.34
Affective Valence	-0.03	-0.05	0.07
Perceived Activation	-0.02	-0.39	-0.34

*Note*. PPA, perceived physical ability; PSPC, physical self-presentation confidence.

## Discussion

This is the first study to examine the psychophysiological responses to group exercise training sessions and to verify whether these responses are intensity-dependent. To examine these objectives, the present research design involved participants performing two group exercise training sessions at different intensities on separate days, which were administered in a counterbalanced order. Specifically, participants were instructed to synchronize their body movements according to music tempo and the trainer’s choreography during moderate (120 beats·min^-1^) and vigorous (145 beats·min^-1^) sessions. As proposed by Karageorghis et al. [17], synchronous music at these tempos may lead participants to exercise at moderate and vigorous intensities, respectively. The group exercise training trials used in this study resulted in different %HRR values (by approximately 15%). Importantly, the %HRR responses to group exercise training trials during both the moderate (from 59% to 79% HRR) and vigorous (from 80% to 90% HRR) sessions were within the exercise intensity range recommended by the ACSM [1] to promote fitness and health benefits (Fig 1). These results are in accordance with the findings from previous studies [10,11] and thereby support the notion of group exercise training sessions as a valuable and effective method to improve health and fitness in active, young adults.

The results of the present study also corroborate with previous findings indicating that vigorous intensities produce less positive affective responses [6,7,14] and demonstrate that the effects of exercise intensity on affective responses are independent of the self-efficacy and self-reported preference for exercise intensity of the participants. These responses are consistent with the fundamental assumptions of the dual mode model [6], which suggest an interplay of cognitive appraisal processes and interoceptive cues in the generation of affective responses during exercise. According to this model, cognitive appraisal processes are the primary determinants of affective responses as the exercise intensity is below or near to the VT. At these exercise intensities, affective responses are pleasant by most people but also unpleasant by many other people. Once the exercise intensity is beyond the VT, interoceptive cues gain salience and become the primary determinant of affective responses. At exercise intensities exceeding the VT, affective responses tend to be homogeneously negative.

The reduction in the affective valence began at the first assessment during exercise and continued until completion of the exercise (Fig 3). Interestingly, there was no significant effect of exercise intensity on post-exercise affect and arousal. Both trials resulted in similar changes in the perceived activation and affective valence immediately after exercise. The reasons for this phenomenon are unclear. However, it is likely that the effects of exercise intensity on affect during exercise are unrelated to affect after exercise. This repeated assessment of “core” affect could be considered a strength of this investigation, as few studies have utilized this methodological approach [31,32]. Additionally, the approach underscores the importance of both assessing and comparing affective responses, both during and after exercise.

The findings of the present study may have implications for exercise prescription in group exercise training programs that differ in intensity. As highlighted, the prescription of vigorous-intensity activities is correlated with less favorable affective responses during exercise. Importantly, the amount of pleasure one experiences during exercise might play a key role in later participation in physical activity [6,5,8]. A more positive affective response experienced during exercise might lead to a greater enjoyment of the activity, creating a positive memory of the exercise experience that may increase the motivation to participate in physical activity in the future. A recent prospective study by Williams et al. [8] confirmed that affective responses to a moderately intense stimulus were predictive of participation in physical activity up to 6 and 12 months later. Thus, interventionists are encouraged to consider that even a very subtle increase in intensity during group exercise training sessions can be enough to make an individual feel less positive about their exercise experience. Given the proposed link between affect and adherence [8], the influence that this "extra" exercise intensity can have on affective responses may be enough to discourage future exercise participation because the experience was less pleasant than one is willing to tolerate.

Although the affective responses experienced during exercise may play a role in predicting adherence to physical activity programs, the exertional responses also have consequences for future physical activity behaviors [8,31]. In the present study, both affective ("how" a person feels) and exertional ("what" a person feels) responses were assessed to provide a more complete picture of the subjective exercise experience. This combination is useful, as the affect and perceived exertion are not isomorphic constructs [23]. As expected, the results from the current study indicate that the vigorous-intensity trial produced more strenuous exertional responses. Additionally, the results demonstrate that the effects of exercise intensity on perceived exertion are unrelated to the self-efficacy and self-reported intensity preference of the participants. Although both trials resulted in similar increases in perceived exertion, the vigorous-intensity trial resulted in a significant increase in effort perception. This increase began at the first assessment during exercise and continued until completion of the exercise (Fig 2). Based on the principles of Borg's [21] model of effort continua, the higher exertional responses reported by the participants during the vigorous-intensity trial are likely to be attributable, at least in part, to the greater physiological requirements of the trial. However, there was no significant effect of exercise intensity on post-exercise perceived exertion. Both trials resulted in similar changes in perceived exertion immediately after exercise. The reasons for this “rebound” effect in the temporal dynamics of the exertional responses are unclear, but the influences of cognitive-social factors [33] and dissociative cues [17] may be plausible explanations. Further research is warranted to elucidate how specific cognitive-social factors and associative/dissociative cues affect exertional and affective responses to group exercise training sessions.

## Limitations

In interpreting the results of this study, readers should take into account its inherent limitations. The sample is the primary limitation. The current sample is rather narrow in terms of age, fitness level, weight status, and socio-educational status. On the one hand, this cohort may not be representative of the adult population at large. On the other hand, this population is appropriate for a preliminary exploration of the effects of exercise intensity on psychophysiological responses to group exercise training sessions. Therefore, the present findings cannot be applied to older adults or individuals with chronic diseases. Similar research with more diverse populations is needed. Secondly, this study did not control for the effects of dispositional variables (extraversion, neuroticism, behavioral inhibition) on affective valence and perceived exertion [33]. It is likely that a degree of the observed variation in the psychophysiological responses to group exercise training sessions may be accounted for by dispositional variables20.

## Conclusions

In summary, this study has provided evidence that psychophysiological responses to group exercise training sessions are intensity-dependent, regardless of differences in self-efficacy beliefs and intensity preferences. Specifically, a moderate-intensity session resulted in lower exercise intensity and perceived activation, less strenuous perceived exertion, and more pleasant affective valence when compared with a vigorous-intensity session performed by active, young adults. From a theoretical perspective, the results of this study provides useful insight into how exercise intensity may influence the associated affective and exertional responses. More importantly, from a practical perspective, it appears that individuals report more positive affective and exertional responses during moderate-intensity exercise sessions, which could create a positive memory of the activity and could hopefully lead to increased motivation for future participation in physical activity.

## Implications

The strength of this investigation lies in the implications of the data for professionals involved in exercise prescription. Interventionists can be confident that group exercise training sessions may elicit physiological requirements that fall within the exercise intensity range recommended by the American College of Sports Medicine to promote fitness and health benefits. On the basis of emerging evidence [10,11], it seems appropriate to highlight the potential role of group exercise training programs as a valuable and effective strategy for improving health and fitness in active, young adults. Interventionists can also be confident that group exercise training sessions at moderate intensities are more helpful for maximizing affective responses and minimizing exertional responses than group exercise training sessions at vigorous intensities, regardless of the self-efficacy beliefs or predispositions for certain exercise intensities among the participants. Such recommendations support the growing sentiment that vigorous-intensity exercise may negatively affect future participation in physical activity [8,14]. That is, vigorous intensities may elicit less pleasant exercise experiences, discouraging the continuation of participation in such strenuous activities in favor of more moderate intensities that result in more positive experiences.
